# A comparison of arsenic exposure in young children and home water arsenic in two rural West Texas communities

**DOI:** 10.1186/s12889-017-4808-4

**Published:** 2017-10-27

**Authors:** Michelle Del Rio, Juan Alvarez, Tania Mayorga, Salvador Dominguez, Christina Sobin

**Affiliations:** 1 Department of Public Health Sciences, College of Health Sciences, University of Texas, 500 W University, El Paso, 79968 USA; 2 The Border Biomedical Research Center, Cancer and Toxicology Core, College of Science, University of Texas, El Paso, USA; 3 Laboratory of Neuroendocrinology, The Rockefeller University, New York, NY USA

**Keywords:** Child arsenic exposure, Child toxicology, Child environmental health

## Abstract

**Background:**

In a previously conducted Health Impact Assessment of a well-water dependent southwest community, arsenic (As) levels greater than the EPA Maximum Contaminant Level (10 μg/L) were identified in home water samples. The goals of this study were to test whether children from the previously studied well-water dependent community (Community 1) had higher blood As levels than children from a demographically similar and geographically nearby community dependent on a municipal water supply (Community 2); to test whether home water As levels predicted child As blood levels; and to examine how child As blood levels changed over time.

**Methods:**

This was an observational study of 252 children aged 4 to 12 years from two communities. Children were recruited through elementary schools and tested during the school day; 204 children participated in follow-up testing. Home water samples were collected according to U.S. Environmental Protection agency recommended procedures. Child heavy metal blood levels and home water sample heavy metal levels were analyzed using inductively coupled plasma mass spectrometry. General linear regression analysis was used to test the influence of community on child As levels, and to examine the contribution of home water As levels to child blood As levels.

**Results:**

Arsenic was detectable in all children tested. Blood levels ranged from 0.09–2.61 μg/dL; approximately 31% of children tested at Time I (79/252) had blood As values above the current acceptable limit (1.2 μg/dL). Approximately 8% of household water samples (6/76) had As levels higher than 10 μg/L. Community did not predict child blood As levels; seasonal effects differed by Community. At Time II, child blood As levels were higher in Community 2 than in Community 1.

**Conclusion:**

A large proportion of children in the communities tested had As exposure. Home water As levels did not predict child blood As levels. Fluctuating child blood As levels by season and over time suggested the contribution of multiple factors and the need for further studies.

## Background

Heavy metal exposure has been associated with many human health consequences including hormone disruption and organ damage, respiratory, metabolic and circulatory disease, and some cancers e.g. [1–10]. Exposure during development to even low level heavy metal is particularly dangerous. As compared to adults, children absorb 40% to 90% more ingested heavy metals [11, 12]. The mechanisms needed to metabolize and eliminate heavy metals evolve throughout childhood [13, 14]. For example, liver pathways that in adulthood metabolize absorbed arsenic do not mature until mid-childhood; un-excreted As continues to circulate and is deposited in other organs [15]. Chronic circulating arsenic burdens and damages organs [16, 17]. Developmental arsenic exposure has also been shown to alter neurodevelopment. In previous studies, child As exposure was associated with lower IQ [18–21]. Another study of low-level As exposure showed inverse linear associations between blood As level and decreased motor function among 303 children aged 8–11 years old [22]. Early disruption of organ systems can increase vulnerability to chronic diseases during adulthood and aging.

Child exposure to arsenic (As) has been of growing concern. An increasing number of reports have identified domestic and public water supply wells with As levels exceeding the Environmental Protection Agency (EPA) maximum contaminant level (MCL) of 10 μg/L [4, 23]; and elevated As levels have been found in children who consumed water exceeding and also below the current standard [24–27]. Moreover, lower detectable child As levels have been associated in several reports with reduced IQ and neurocognitive function [20, 28–33] raising the possibility that current exposure threshold should be lowered.

Geological studies have provided evidence that risk of exposure to As is particularly elevated in the Southwest United States. For example, a comprehensive study of ground water samples reported that 25.8% of southwest regions tested had As concentration levels above the current maximum contaminant level (MCL) of 10 μg/L, with values ranging from 10 to 24 μg/L; an additional 16.9% had levels ≥25 μg/L. Specific regional analysis of the Rio Grande aquifer samples showed that 19.2% were expected to have As levels exceeding 10 μg/L [34].

Given this regional risk, a recent Health Impact Assessment (HIA) was conducted in a rural incorporated village supplied by the Rio Grande Aquifer, dependent on water from privately owned or domestic wells and without access to a municipal water supply [35]. Approximately half of water samples tested had As levels exceeding 10 μg/L. Children living in this village had not been tested for exposure to As (or other heavy metals).

The goal of current study was to test As blood levels in children from the village studied by the previous Health Impact Assessment [35]. We examined whether children living in the village dependent on well-water sources had higher blood As levels as compared to a demographically similar and geographically nearby population of children living in an unincorporated township using the municipal water supply. To test for the possible source of As blood levels, for a subsample of children in each community, home water samples were also tested and used to predict child As blood levels. For comparison, lead (Pb) and cadmium (Cd) levels in child blood and home water samples were also tested. We predicted that children living in the well-water dependent village would have significantly higher blood As levels as compared to children from the township using water from the public system. We also predicted that home water As would predict child blood As levels.

## Methods

### Ethics and consents

Prior to contact with any participating subjects approval for these studies and all of the methods contained herein were approved by the Institutional Review Board of the University of Texas, El Paso (Protocol Ref #564493–3). Parent consent was obtained as detailed below approximately three weeks prior to child testing; child assent was obtained immediately prior to testing.

### Study setting and design

Children from two communities were studied. The two communities from which children were recruited included one incorporated village dependent on well-water with no access to a municipal water supply (Community 1) and one unincorporated township located approximately 2 miles to the east of the village, whose water source was a municipal water supply (Community 2). Because children in these communities had not been tested previously for heavy metal exposure, effect and sample size estimations were not attempted, and as many children as possible were recruited from each community.

### Study population

All children in this study lived and attended elementary school in one of two communities located approximately 20 miles north of the US-Mexico border. Community 1 had an estimated 1529 residents and 408 households, with 52% of households having 4 or more persons of predominantly Hispanic or Latino descent (96.1%), and including 316 children between the ages of 5 and 14 [36]. All families in this community depended on water from domestic wells or privately owned public wells.

Community 2 was an unincorporated township located approximately 2 miles east of Community 1 and separated from it by a major interstate highway. Community 2 had an estimated 3938 residents and 992 households, with 56.9% of the households having 4 or more persons of predominantly Hispanic or Latino descent (98.9%), and including 667 children between the ages of 5 and 14 years [36]. All families in Community 2 depended on water from the local municipal water system.

### Recruitment and sample

Parents and children were recruited from the elementary school located in Community 1 and Community 2 through notification letters from the school principals and during Parent-Teacher Conference nights. Children included for study were 4 to 12 years of age and up to two siblings per family were allowed to participate. When more than two siblings were available for participation, the two youngest siblings were included. Parent informed consent for child participation was obtained at the time of recruitment. Researchers communicated and followed up with parents throughout the study via school-approved forms and notes taken home to parents by participating children. All study forms and all study materials were given in Spanish and English versions. Researchers working on this study were bilingual and throughout the study interacted with children and parents in the participant’s preferred language.

The numbers of children recruited from the two communities were roughly proportional to the estimated number of eligible children in each community (see above). A total of 102 children completed Time I testing from Community 1 and 150 children completed Time I testing from Community 2. Child assent was obtained immediately prior to testing. With regard to Community 1, of 112 children recruited, 5 children left the school before data collection was completed, 4 children declined the finger-stick blood collection procedure and 1 child was absent during study participation days. With regard to Community 2, of 158 children recruited, 6 children left the school before data collection began and 2 children declined the finger-stick blood collection procedure.

Child As values from the first group recruited and testing April to May (Community 1, *n* = 44; Community 2, *n* = 74), prompted us to recruit and test additional children approximate 5 months later October to November (Community 1, *n* = 58; Community 2, *n* = 76) and also re-test all children during the following April and May (an average of nine months following the first testing). The variable “Group” indicated when children were recruited and was included in all regression models.

Funding allowed household water sample testing immediately after Time I child testing for a subsample of 118 recruited children in Communities 1 and 2 from 84 households. The 84 households represented all of the households for the 118 recruited children. Of the 84 consenting households, 74 home water samples were collected, 31 from Community 1 and 43 from Community 2. Two missing samples were able to be collected from next-door neighbors who shared the same water supply, yielding 33 samples from Community 1 and 43 samples from Community 2. For the regression model predicting child As blood level from household water As level, 8 missing values for home water As level were replaced with the community As home water sample mean value. The attitude of parents towards household water testing was positive; the inability of households to provide water samples was generally because of communication difficulties (no phone) or conflicting work schedules.

Time II testing was conducted an average of nine months after Time I testing. As indicted in Table 1, 81% of children (204/252) completed Time II testing, which was conducted during one two-week interval. The remaining 19% of children who did not complete Time II testing (48/252) were lost to follow up due to geographic relocation of families.

**Table 1:** Demographic characteristics of participants by community at Time I and Time II

	Time I (*N* = 252)			Time II (*N* = 204)		
Variable	Community 1	Community 2	Total	Community 1	Community 2	Total
*N*	102	150	252	87	117	204
Age, *M*(SD)	8.19 (± 1.86)	7.89 (± 1.95)	8.01 (± 1.92)	8.67 (±1.83)	8.50 (±1.87)	8.57 (±1.85)
Females %	42.2%	56.0%	50.4%	37.9%	53.8%	47.1%
Mother’s Ethnicity *n* (%)	72/102 (71%)	115/150 (77%)	187/252 (74%)	59/87 (68%)	91/117 (78%)	150/204 (74%)
Hispanic/Mexican-American	98.6%	98.3%	98.4%	98.3%	100.0%	99.3%
White	1.4%	1.7%	1.6%	1.7%	0.0%	0.7%
Father’s Ethnicity *n* (%)	70/102 (69%)	109/150 (73%)	179/252 (71%)	57/87 (66%)	88/117 (75%)	146/204 (72%)
Hispanic/ Mexican-American	100.0%	93.6%	96.1%	100%	94.3%	95.9%
African American	0.0%	1.80%	1.1%	0.0%	1.1%	0.7%
Pacific Islander	0.0%	0.9%	0.6%	0.0%	0.0%	0.0%
White	0.0%	3.7%	2.2%	0.0%	4.5%	2.7%
Mother’s Education *n* (%)	73/102 (72%)	115/150 (77%)	188/252 (75%)	60/87 (69%)	91/117 (78%)	151/204 (74%)
Completed grades 1–6	5.9%	12.2%	10.6%	8.3%	14.3%	11.9%
Some high school	9.8%	20.9%	18.1%	16.7%	19.8%	18.5%
Graduated high school	30.4%	18.3%	27.7%	40.0%	17.6%	26.5%
Some college	19.6%	40.9%	35.7%	31.7%	39.6%	36.4%
Graduated college	2.9%	6.1%	5.3%	3.3%	6.6%	5.3%
Some graduate school	2.0%	0.0%	1.1%	0.0%	0.0%	0.0%
Completed graduate school	0.9%	0.0%	0.5%	0.0%	1.1%	0.7%
More than graduate school	0.0%	0.9%	0.5%	0.0%	1.1%	0.7%
Other (Certification)	0.0%	0.9%	0.5%	0.0%	0.0%	0.0%
Household Income *n* (%)	61/102 (60%)	97/150 (65%)	158/252 (63%)	49/87 (56%)	76/117 (65%)	125/204 (61%)
Median	$18,000	$18,000	$18,000	$18,000	$18,500	$18,000
Household Family Size *n* (%)	74/102 (73%)	116/150 (77%)	190/252 (75%)	61/87 (70%)	92/117 (79%)	153/204 (75%)
*M* (SD)	5.23 (±1.41)	5.14 (±1.44)	5.17 (± 1.42)	5.31 (±1.48)	5.20 (±1.50)	5.24 (±1.49)

Parents completed a family and child demographic and health assessment form that included questions on family composition, ethnicity, education, income and child development including medical, cognitive and behavioral information, and possible history of heavy metal testing. No remarkable medical, cognitive, and/or behavioral diagnoses were reported by parents for this sample of children; only one child had been previously tested for lead exposure with negative results. All testing took place during the regular school day. Notes sent home to consented parents of participating children reminded them one day prior to testing. On the day of testing, small groups of participating children were brought to the study testing room during their Physical Education class time. Families were compensated for participation with a five-dollar gift card to a local retail store after completion of each study phase (form completion, child testing, and water sample collection). All study results were shared and discussed with parents during small group reporting sessions and community dinners held at the elementary schools that school personnel attended.

### Blood sample collection and analysis

After arriving in the testing room, child assent was obtained and children’s hands were first cleaned with a liquid hand cleaner, washed thoroughly with soap and water and dried with paper towel. Testers then cleaned the sample finger (usually ring-finger of the left hand) with an alcohol swipe before lancing. One 50 μl whole blood finger stick sample was collected from each child into a sterile EDTA micro-vial via capillary tubes, stored at 4 °C and later transferred to the chemistry laboratory at New Mexico State University, Las Cruces (Tanner Schuab, PhD, laboratory head) for analysis by inductively-coupled plasma mass spectrometry (ICP-MS) (arsenic, lead, and cadmium). All metal levels were recorded in micrograms per deciliter (μg/dL).

### Anthropometrics

Height, weight, blood pressure (Sphygmomanometer Model 08A, Contec Medical Systems, Qinhuangdao, China), and waist and hip circumference, were measured for all children.

### Home water sample collection and analysis

Permission to collect home water samples was included in the original informed consent. Appointments were scheduled. Household water samples were collected according to U.S. Environmental Protection Agency procedures [37]. On the day of sample collection, a two-member research team collected a kitchen faucet sample and completed a 19-item household water practice use survey (not the focus of the current analyses). Water sampling consisted of identifying the kitchen faucet, removing faucet mouth filter, cleaning the faucet mouth with 70% alcohol and lint-free wipes, and completing a five second ambient temperature water run. The water sample was then collected into a sterile beaker. The pH level and water temperature were measured using a portable electronic pH meter (Thermo-Scientific Orion 5-Star, Fisher Scientific, Waltham, MA) and recorded. From the water sample, approximately 250 mL was poured into a screw top plastic container treated with 2% nitric acid (prepared containers provided by the analyzing laboratory, Alamo Analytical Laboratories, San Antonio, TX). The sample was labeled and stored at approximately 4C° and transported the following day to the analyzing laboratory (local office of the home laboratory). Home water samples were analyzed by ICP-MS for heavy metals (arsenic, lead, and cadmium) and values were recorded in μg/dL.

### Statistical analyses

Descriptive and inferential statistics were analyzed with *SAS Version 9.3.* (SAS Institute Inc., Cary, North Carolina) and *SPSS Version 23* (IBM, Armonk, New York). Data were entered, checked for accuracy, and examined by community for missing values and distribution properties. For the main hypotheses, regression models tested whether child As levels differed at Time I or Time II by Community; whether home water sample As level predicted child As blood level; and whether child As blood level changed significantly from Time I to Time II (repeated measure) and/or differed by community. Prior to conducting the main analyses, regression was also used to test for differences from established child exposure thresholds for three heavy metals tested (one model); to determine whether sex or age or the interaction predicted child As level; and to determine whether home water As differed by community. F and *p* values associated with Type III sum of squares were interpreted for all models. Given the total number of models calculated, statistical significance was evaluated using *p* ≤ .01.

Skewness estimates for child blood As distributions were 0.72 at Time I and 2.01 at Time II. General linear models are relatively robust to violations of normality, particularly for larger samples sizes, e.g. *N* > 50. Nonetheless, to test this robustness, values 1.5 times greater than the upper quartile were identified as outliers and excluded (5/252 cases at Time I; 2/204 cases at Time II). With outliers excluded, general linear models predicting child As blood level at Time I and Time II were re-calculated. Excluding outliers resulted in no change in model results. The results of linear models excluding outliers are provided in parentheses in the text following each report of general linear model results including all cases. Geometric mean values for child blood As Time I and Time II are provided in Tables 2 and 6.

**Table 2:** Anthropometrics and heavy metal blood levels of children at Time I, *N* = 252

	Time I		
Variable	Community 1	Community 2	Total
n	102	150	252
Height (in) M (SD) Range	50.37 (± 5.34) 38.39–62.24	49.86 (± 5.53) 36.89–61.22	50.07 (± 5.45) 36.89–62.24
Weight (lbs) M (SD) Range	66.77 (± 24.76) 26.40–146.00	66.89 (± 26.89) 29.40–153.60	66.84 (± 25.99) 26.40–153.60
BMI^a^ *n* (%)	99/102 (97%)	149/150 (99%)	251/252 (99%)
*Underweight (< 5th %ile)*	5.1%	2.7%	3.6%
*Normal (5th–85th %ile)*	65.7%	62.4%	62.2%
*Overweight or Obese (≥ 85th %ile)*	29.3%	36.2%	33.1%
*Obese (*≥ *95th %ile)*	16.2%	22.1%	19.5%
Heavy metal (μg/dL)			
As M (SD) Range	0.89 (± 0.57) 0.09–2.61	1.03 (± 0.38) 0.42–2.52	0.97 (± 0.47) 0.09–2.61
Pb M (SD) Range	0.84 (± 0.96) 0.00–5.29	1.03 (± 0.79) 0.12–4.39	0.95 (± 0.87) 0.00–5.29
Cd M (SD) Range	0.05 (± 0.05) 0.00–0.32	0.07 (± 0.08) 0.00–0.49	0.07 (± 0.07) 0.00–0.49

## Results

The demographic characteristics of the analyzed sample of 252 children are shown in Table 1.

Demographic and health and personal history data were collected at Time I (*N* = 252); the distributions of these characteristics at both time points (Table 1) show minor shifts in the demographic composition of the sample from Time I to Time II (*N* = 204). The sample included approximately equal numbers of males and females with a mean Time I age of 8.01 (±1.92). Approximately 98% of parents self-identified as Hispanic, Latino, Mexican-American, or Mexican. Family median annual household income was approximately $18,000. The mean family size was 5.17 (±1.42). For households with five children below the age of 18, the U.S. national poverty threshold was $28,695 (U.S. Census 2014).

### Child heavy metal blood levels

Table 2 summarizes child anthropometric measurements and heavy metal blood levels.

**Fig. 1:**
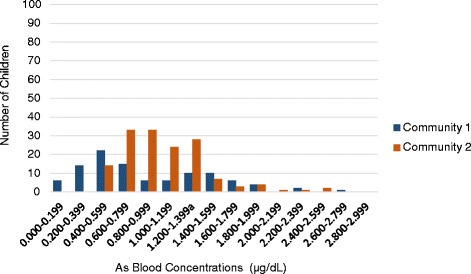
Frequency of child As blood levels at Time I by community (*N* = 252)

Consistent with national averages, approximately 33% of the children met criteria for overweight or obese according to age and sex adjusted body mass indices. With regard to heavy metal exposure, the current allowable exposure limits are 1.2 μg/dL for As (children and adults) [38–40]; 0.50 μg/dL for Cadmium (Cd) (children and adults) [41] and 5 μg/dL for lead (Pb) (child-specific level for “elevated”) [42]. As shown in Table 2, at Time I, the mean child blood As level in both communities approached the exposure limit while mean Cd and Pb levels were well below current exposure thresholds (Time I As geometric mean = 0.85). As Table 3 and Fig. 1 illustrate, As levels ranged from 0.09 to 2.61 μg/dL; approximately 31% of children (79/252) had blood As values above the defined limit. An additional 12% of children (30/252) had borderline levels between 1.000–1.199 μg/dL.

**Table 3:** Frequencies of child blood As levels at Time I testing by community (*N* = 252)

	Community 1				Community 2			
As intervals (μg/dL)	Freq	Freq %	Cumul Freq	Cumul Freq %	Freq	Freq %	Cumul Freq	Cumul Freq %
2.800–2.999	0	0.0	102	100.0	0	0.0	150	100.0
2.600–2.799	1	1.0	102	100.0	0	0.0	150	100.0
2.400–2.599	0	0.0	101	99.0	2	1.3	150	100.0
2.200–2.399	2	2.0	101	99.0	1	0.7	148	98.7
2.000–2.199	0	0.0	99	97.1	1	0.7	147	98.0
1.800–1.999	4	3.9	99	97.1	4	2.7	146	97.3
1.600–1.799	6	5.9	95	93.1	3	2.0	142	94.7
1.400–1.599	10	9.8	89	87.3	7	4.7	139	92.7
1.200–1.399^a^	10	9.8	79	77.5	28	18.7	132	88.0
1.000–1.199	6	5.9	69	67.6	24	16.0	104	69.3
0.800–0.999	6	5.9	63	61.8	33	22.0	80	53.3
0.600–0.799	15	14.7	57	55.9	33	22.0	47	31.3
0.400–0.599	22	21.6	42	41.2	14	9.3	14	9.3
0.200–0.399	14	13.7	20	19.6	0	0.0	0	0.0
0.000–0.199	6	5.9	6	5.9	0	0.0	0	0.0
Total	102	100.0	102	100.0	150	100.0	150	100.0

^a^ *1.2 μg/dL, CDC As blood level limit*

### Heavy metal exposure comparison

**Fig. 2:**
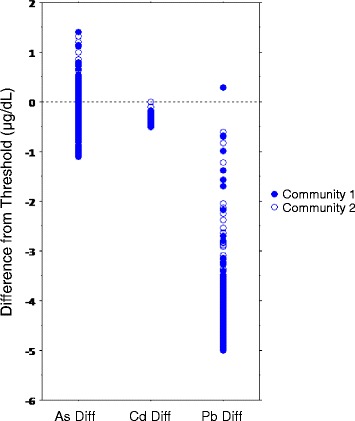
Child heavy metal value difference from threshold for arsenic, cadmium and lead (*N* = 252)

Before the main analyses were conducted general linear regression was used to examine whether exposure levels relative to recommended limits differed in children among the heavy metals tested. The difference from threshold for each metal level was calculated and the values were compared across the heavy metals assessed (each child contributed three difference values). Substantially more children had As blood levels exceeding the As threshold as compared with Cd and Pb (model F_2,753_ = 3546.55, *p* < .001; adj R-square = 0.90, MSE = 0.33, CV = 36.49). Fig. 2 illustrates the difference. Thus, as compared to Cd and Pb, children in this sample were differentially exposed to As. No further models examining Cd or Pb were conducted.

### Influence of sex and age on as blood level

To test possible control factors needed for the main analyses general linear regression was used to determine whether sex, age or the interaction of sex and age, predicted child blood As level at Time I or Time II. The overall models were not statistically significant for Time I (model F_3,248_ = 1.04, *p* = 0.38; adj R^2^ = 0.001, MSE = 0.22, CV = 48.68); or Time II (model F_3,199_ = 0.67, *p* = 0.61, adj R^2^ = −0.01, MSE = 0.06, CV = 57.37) (with outliers excluded, Time I model F_3,243_ = 0.90, *p* = 0.41; adj R^2^ = − 0.001; Time II model F_3,197_ = 1.56, *p* = 0.21; adj R^2^ = 0.006). Sex and age were not included in further models examining child As levels.

### Comparison of child blood arsenic levels by community

General linear regression was used to test whether Community predicted child blood As levels at Time I, controlling for recruitment Group and the interaction of Community x Group. The overall model was significant (model F_3,248_ = 85.70, *p* < 0.001; adj R-square 0.50, MSE = 0.11, CV = 34.32) (with outliers excluded, model F_3,243_ = 92.34, p < 0.001; adj R^2^ = 0.53). Table 4 shows the regression model change statistics, estimated standardized coefficients, and 95% confidence intervals for the beta estimates.

**Table 4:** Association of community and child As blood level controlling for recruitment group (*N* = 252)

	Model change statistics						Full model coefficient statistics			
	Adj R^2^	SE	R^2^ Change	F	df	p	B (SE)	t	p	95% C.I.
Community	0.02	0.47	0.02	5.41	1/250	0.021	−1.48 (0.22)	−6.61	<0.001	−1.91 /−1.04
Group	0.40	0.37	0.38	161.77	2/249	<0.001	−5.30 (0.66)	−8.07	<0.001	−6.59 /− 4.01
Community x Group	0.50	0.33	0.10	51.67	3/248	<0.001	0.62 (0.09)	7.19	<0.001	0.45 /0.79

**Fig. 3:**
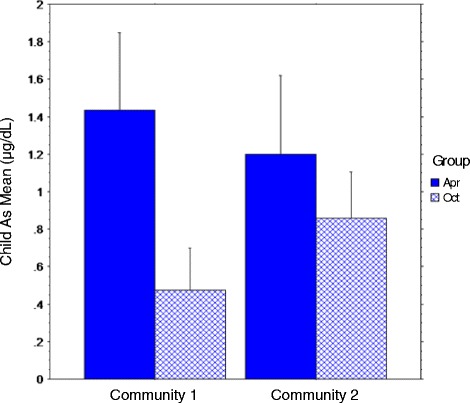
Significant interaction of Community by Group for Time I child blood As level with standard deviation bars (N = 252)

As suggested by the small adjusted R^2^ for Community (Table 4), the sum of squares was not significant for Community (F = 2.96, *p* = 0.09). Sum of squares for Group (F = 228.25, p < 0.001) and for Community x Group (F = 51.69, p < 0.001) were significant. The interaction is illustrated in Fig. 3. Group 1 was recruited and tested in April and May; Group 2 was recruited and tested in October and November. Thus, child blood As levels appeared to be influenced by the season in which samples were drawn and tested, with opposite effects by community. For children in Community 1, child As blood levels were lower in fall as compared to spring; in Community 2, child As blood levels were higher in fall as compared to spring.

### Home water as analyses

The levels of heavy metals in household water samples are shown in Table 5.

**Table 5:** Home water sample heavy metal levels by community

	Time I		
Variable	Community 1	Community 2	Total
*N*	44	74	118
Heavy Metal M (SD) Range			
As (μg/L)	7.1 (± 0.33) 0.00–16.00	3.7 (± 0.32) 0.00–10.0	0.50 (± 0.36) 0.00–1.60
Pb (μg/L)	0.0024 (± 0.02) 0.00–0.10	0.00 (± 0.00) 0.00–0.00	0.009 (± 0.009) 0.00–0.10
Cd (μg/L)	0.01 (± 0.09) 0.00–6.00	0.22 (± 1.02) 0.00–8.70	0.14 (± 0.82) 0.00–8.70
pH	7.76 (± 0.38) 7.09–8.21	7.28 (± 0.14) 6.39–7.53	7.46 (± 0.35) 6.39–8.21

Approximately 8% of household water samples (6/76) had As levels higher than 10 μg/L (current EPA MCL), and the highest level in household water assessed was 16 μg/L. All of the households with higher than acceptable As levels were in Community 1 (well-water dependent community). Similar to child blood levels, the levels of lead and cadmium in household water (Table 5) were consistently below the current MCLs (MCL for lead in drinking water is 15 μg/L; MCL for cadmium in drinking water is 5 μg/L) [42].

Home water heavy metal analysis was conducted for households of children in only the first recruitment group (i.e., group was not a variable in this regression). Community differences in home water pH levels (Table 5) were noted and single-order correlation showed that Community and home water pH were positively correlated (*r* = 0.43). A regression analysis was conducted to test whether home water As level was predicted by Community; given the correlation between Community and home water pH, pH was not included as a predictor. The overall model was significant (F_1,74_ = 22.25, *p* < .001; adj R^2^ = 0.22; MSE = 0.12; CV = 68.23), and sum of squares for Community was significant (F = 22.25, p < 001). Corroborating findings from the original Health Impact Assessment [35], home water As levels of Community 1 were higher than Community 2.

**Fig. 4:**
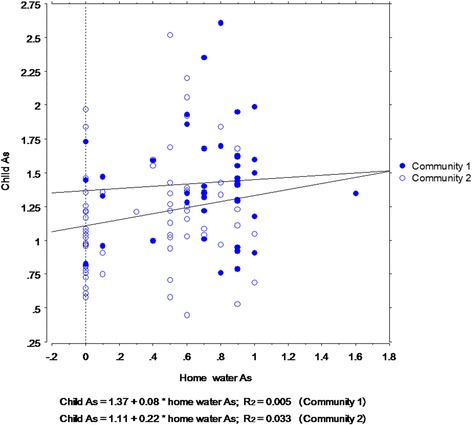
Association between child blood As and home water As by Community (*N* = 118)

General linear regression was used to predict child blood As level from home water sample As level, controlling for Community. The overall model was significant however the amount of variance explained was small (model F_3,105_ = 3.62, *p* = 0.016; adj R^2^ = 0.07, MSE = 0.16, CV = 31.60) and sum of squares for the predictors was not statistically significant (home water As level, F = 0.25, *p* = 0.69; Community, F = 2.43, *p* = 0.12; home water As x Community, F = 0.35, *p* = 0.56). The slopes are shown in Fig. 4. While detectable levels of As were found in home water samples from both communities, and home water As was significantly higher in Community 1, child blood As levels were not attributable to home water sample As levels. (Child As outlier values were not among those with home water sample testing.)

### Child as blood levels over time

Follow-up testing was conducted among 204 children available for participation at Time II. Table 6 shows the anthropometrics and mean child heavy metal blood levels for As, Pb and Cd at Time II testing.

**Table 6:** Blood levels and anthropometric measures of children at Time II (*N* = 204)

	Time II		
Variable	Community 1	Community 2	Total
N	87	117	204
Anthropometric n (%)	87/87 (100%)	115/117 (98%)	202/204 (99%)
Height (in) M (SD) Range	51.48 (± 5.27) 39.02–63.70	51.43 (± 5.38) 37.32–62.36	51.45 (± 5.32) 37.32–63.70
Weight (lbs) M (SD) Range	70.58 (± 25.26) 28.20–114.6	72.19 (± 28.76) 29.80–180.40	71.50 (± 27.25) 28.20–180.40
BMI^a^ *n* (%)	85/87 (98%)	115/117 (98%)	200/204 (98%)
*Underweight (< 5th %ile)*	6%	3%	5%
*Normal (5th–85th %ile)*	67%	61%	64%
*Overweight or Obese (≥ 85th %ile)*	27%	36%	32%
*Obese (*≥ *95th %ile)*	15%	20%	18%
Heavy Metal (μg/dL)			
As M (SD) Range	0.34 (± 0.18) 0.03–1.21	0.47 (± 0.28) 0.05–2.07	0.43 (± 0.25) 0.03–2.07
Pb M (SD) Range	0.85 (± 0.65) 0.20–3.93	0.94 (± 0.56) 0.02–4.17	0.90 (± 060) 0.02–4.17
Cd M (SD) Range	0.04 (± 0.04) 0.00–0.32	0.05 (± 0.05) 0.00–0.25	0.05 (± − 0.05) 0.00–0.32

^a^ *Based on age and sex norms*

**Fig. 5:**
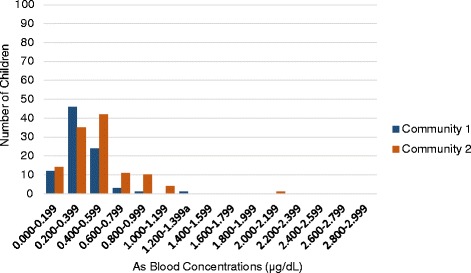
Frequency of child As blood levels at Time II, by community, *N* = 204

**Fig. 6:**
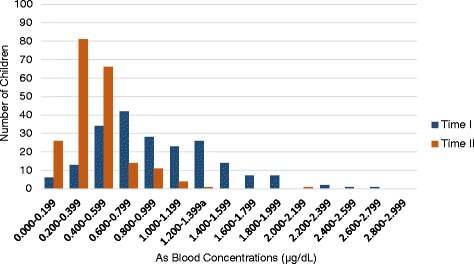
Frequency of Children As Blood Levels Comparing Time I and Time II Tests, *N* = 204

Table 7 and Fig. 5 show the distribution of child As levels at Time II by Community. Table 8 and Fig. 6 compare the blood As distributions at Time I and Time II for 204 children who completed testing at both time points.

**Table 7:** Frequencies and cumulative frequencies of child blood As levels at Time II testing by community (*N* = 204)

	Community 1				Community 2			
As intervals (μg/dL)	Freq	Freq %	Cumul Freq	Cumul Freq %	Freq	Freq %	Cumul Freq	Cumul Freq %
2.800–2.999	0	0.0	0	0.0	0	0.0	0	0.0
2.600–2.799	0	0.0	87	100.0	0	0.0	117	100.0
2.400–2.599	0	0.0	87	100.0	0	0.0	117	100.0
2.200–2.399	0	0.0	87	100.0	0	0.0	117	100.0
2.000–2.199	0	0.0	87	100.0	1	0.9	117	100.0
1.800–1.999	0	0.0	87	100.0	0	0.0	116	99.1
1.600–1.799	0	0.0	87	100.0	0	0.0	116	99.1
1.400–1.599	0	0.0	87	100.0	0	0.0	116	99.1
1.200–1.399^a^	1	1.1	87	100.0	0	0.0	116	99.1
1.000–1.199	0	0.0	86	98.9	4	3.4	116	99.1
0.800–0.999	1	1.1	86	98.9	10	8.5	112	95.7
0.600–0.799	3	3.4	85	97.7	11	9.4	102	87.2
0.400–0.599	24	27.6	82	94.3	42	35.9	91	77.8
0.200–0.399	46	52.9	58	66.7	35	29.9	49	41.9
0.000–0.199	12	13.8	12	13.8	14	12.0	14	12.0
Total	87	0.0	87	100.0	117	100	117	100.0

^a^ *1.2 μg/dL allowable limit of As blood levels in children set by the CDC*

**Table 8:** Frequencies of child blood As levels at Time I and II (*N* = 204)

Time I					Time II			
As range (μg/dL)	Freq	Freq %	Cumul Freq	Cumul Freq %	Freq	Freq %	Cumul Freq	Cumul Freq %
2.800–2.999	0	0.0	204	100.0	0	0.0	204	100.0
2.600–2.799	1	0.5	204	100.0	0	0.0	204	100.0
2.400–2.599	1	0.5	203	99.5	0	0.0	204	100.0
2.200–2.399	2	1.0	202	99.0	0	0.0	204	100.0
2.000–2.199	0	0.0	200	98.0	1	0.5	204	100.0
1.800–1.999	7	3.4	200	98.0	0	0.0	203	99.5
1.600–1.799	7	3.4	193	94.6	0	0.0	203	99.5
1.400–1.599	14	6.9	186	91.2	0	0.0	203	99.5
1.200–1.399^a^	26	12.7	172	84.3	1	0.5	203	99.5
1.000–1.199	23	11.3	146	71.6	4	2.0	202	99.0
0.800–0.999	28	13.7	123	60.3	11	5.4	198	97.1
0.600–0.799	42	20.6	95	46.6	14	6.9	187	91.7
0.400–0.599	34	16.7	53	26.0	66	32.4	173	84.8
0.200–0.399	13	6.4	19	9.3	81	39.7	107	52.5
0.000–0.199	6	2.9	6	2.9	26	12.7	26	12.7
Total	204	100.0	204	100.0	204	100.0	204	100.0

^a^ *1.2 μg/dL, CDC allowable As blood limit*

Overall, child blood As levels decreased over time (Time II geometric mean = 0.37 μg/dL). General linear regression was used to examine whether Community predicted child blood As levels at Time II. The overall model was statistically significant (F_1,202_ = 8.51, *p* = 0.004; adj R^2^ = 0.04; MSE = 0.06; CV = 56.15) (with outliers excluded, F_1,200_ = 10.62, *p* = .001; adj R^2^ = 0.05) and accounted for a small but significant amount of variance in child As levels. At Time II, and contrary to our predictions, child As blood levels were higher in Community 2 as compared to Community 1.

General linear regression was also used to test As blood level change over time including one repeated measure (child As level, Time I/Time II) and one predictor (Community). MANOVA test criteria were significant only for child As blood level (Wilk’s lambda F_1,202_ = 203.95, *p* < .001); the interaction of child As blood level and Community was not significant (Wilk’s lambda F_1,202_ = 0.59, *p* = 0.45). For children from both communities, As blood levels decreased from Time I to Time II testing.

## Discussion

This study followed on a formal Health Impact Assessment showing elevated As levels in source well-water supplies of a rural village in the U.S. Southwest [35], and aimed to determine whether children in the well-water supplied village (“Community 1”) had elevated blood As levels as compared to children living in a geographically nearby and demographically similar town using a municipal water supply (“Community 2”). The study used ICP-MS for all blood and water sample analysis, a reliable and valid method for accurate detection of low level heavy metals. ICP-MS analysis of blood for determining child As exposure has some advantages over the detection of As in urine. Urine concentration measures excreted levels of As, and to some extent, these are influenced by child hydration which can fluctuate substantially in dry climates such as the high desert southwest [43]; blood as compared to urine levels indicate tissue burden [38]. A preliminary regression analysis showed that as compared to lead and cadmium, children in the communities studied were differentially exposed to As. This is consistent with previous studies showing heightened risk in the U.S. Southwest for As in ground water [34].

There was a broad range of low elevated As exposures in both communities at Time I, and at Time I, a large proportion of children from both communities were found to have As levels above the current acceptable limit (1.2 μg/dL). Contrary to prediction, community did not predict child As blood levels at Time I. At Time II, low detectable As blood levels were also observed but were lowered overall as compared to Time I; and at Time II, blood As levels were higher in Community 2.

There is broad recognition that no level of As exposure is “safe” during development. (While 10 μg/L is the stated EPA water supply “limit,” the MCL goal is 0.) Child blood thresholds are often set according to an estimated percentage of children or adults with a given level of exposure based on aggregated data, e.g., the current 5 μg/dL criteria for “elevated” blood lead in children represents the estimated blood lead level of less than 5% of U.S. children between the ages of 0 to 5 years. While this provides a reasonable starting point, the meaning of a “threshold” determined by estimated percentages of children exposed, is arbitrary with regard to child outcomes. Instead, a future goal must be evidence-based limits that indicate what levels of exposure over what period of time, predict lasting effects on physical and mental health.

With regard to the findings of the current study, an alarming number of children in these low-income communities had detectable levels of As at both time points. The change in number of children below the current “limit” should not suggest that the population of children went from danger to no danger. As is more mobile than other heavy metals (e.g., lead) and leaves the body relatively quickly, usually within approximately 2 days. The change in values over time suggested that studies are needed to determine the frequency of monitoring required and to better understand the correspondence between exposure and blood level evidence of exposure, and how exposure fluctuations may alter outcome.

Home water samples were also analyzed to determine whether a likely source of child exposure was from the home water supply. Consistent with our hypotheses, as compared to homes from Community 2, homes from Community 1 had significantly higher As levels. Home water As however did not predict child As blood levels. If the primary exposure source was from home tap water, given the relatively rapid mobility of As, fluctuating levels of water As might be expected to produce fluctuating child blood As levels. The small association suggests that additional studies are needed to repeatedly measure home and child As levels in both blood and urine to determine the strength of the correspondence over time. At this time we concluded that home drinking water supplies alone were an unlikely major source of child exposure.

There are many other plausible exposure sources for these children. Ingestion of heavy metals from soiled hands is believed to be a primary exposure source in children when As is present in residential soil, household dirt or dust [38, 44]. Children’s diets can also create risk. Relative to body size, children consume larger proportions of foods that commonly carry As such as rice, apples and apple products, and some vegetables [4, 45, 46]. Low-income children may be at special risk due to poor diet lacking in essential nutrients like calcium, zinc, iron, and vitamin C that would otherwise help to inhibit heavy metal absorption [47–49].

There was no obvious explanation for changes in As values from Time I to Time II however there are many possibilities. In the high desert southwest wind events in the spring and subsequent dust storms can distribute air-borne heavy metal particles and nanoparticles from historically contaminated soil. Temperature extremes during summer months might also change the concentrations of As in water sources. A local metals processing plant that was determined to be out of regulatory compliance approximately 10 years prior to when this study was conducted, and located between these two communities, may have been responsible for historical contamination that continued to create random exposure events due to a combination of factors such as local construction requiring large-scale soil exchange and removal, high winds, air pollution patterns over the interstate that separates the communities studied and differences in the amount of time children spend outdoors. (Additional studies of the local soil, air, and rice supplies from community markets were undertaken following the first phase of testing to help these communities identify possible sources of child As exposure and will be separately reported.) The interaction of child As blood level and season of testing (spring vs. fall) is also important to consider in this context. For the well-water dependent community, child As levels were higher in spring and lower in fall; for the municipal water supply dependent community, As levels were lower in the spring and higher in fall. This interaction may point to differences between communities with regard to the influence of season-relevant effects. Studies are underway to explore these possibilities.

Home water As levels were significantly higher in the well-water dependent community (Community 1), confirming findings from the initial HIA. At the same time, and contrary to our hypothesis, source well-water was not a significant predictor in the models tested. Thus for these communities, providing access to the municipal public water system appeared to reduce the risk of As in the home water, but not in the child. The interaction of testing season and community also seemed to suggest that differences observed were not due to the community water source.

It was noted that as compared to Community 2, home water pH level in Community 1 was significantly higher, and pH was a single predictor of home water As level (positively correlated, *r* = 0.43). This finding was consistent with previous studies conducted in the U.S. southwest and other U.S. regions [23, 34]. Increasing alkalinity increases the solubility of As containing source minerals such as iron oxides and sulfides, thus increasing the release of As [23]. Logically, one might expect that controlling the alkalinity of water could reduce As levels. Maintaining a constant water pH level however is difficult to achieve. A more feasible solution may be the removal of source minerals from the water using appropriate filters. While public water systems use filtration systems, the As levels in the home water samples from Community 2 suggested that more specialized approaches may be needed.

## Conclusions

In over 200 children from demographically similar communities in the southwest U.S., As exposure was common. Source well-water, an assumed risk factor in southwestern rural regions, was not a significant contributor to child blood As level. As in blood samples was analyzed to estimate tissue burden. Given the mobility of As in children’s bodies however the findings suggested that characterization of child As exposure will require studies that simultaneously measure As in blood, urine, and environmental air, water and soil samples in order to narrow the possible sources of exposure. Exposure to As during development is expected to have markedly different effects than exposure during adulthood and the effects of chronic exposure on developing systems may increase vulnerability to chronic disease in adulthood and aging. Studies are also needed to establish child-relevant evidence-based maximum contaminant levels that reflect risk of specific short- and longer-term outcomes to the developing child.

## Abbreviations

As: Arsenic
ASTDR: Agency for toxic substances and disease registry
Cd: Cadmium
CDC: U.S. center for disease control
EDTA: Ethylenediaminetetraacetic acid
EPA: U.S. environmental protection agency
HIA: Health impact assessment
ICP-MS: Inductively coupled plasma mass spectrometry
IQ: Intelligence quotient
IRB: Institutional Review Board
MCL: Maximum contaminant level
Pb: Lead
